# Electrochemical Polishing of Ti and Ti_6_Al_4_V Alloy in Non-Aqueous Solution of Sulfuric Acid

**DOI:** 10.3390/ma17122832

**Published:** 2024-06-10

**Authors:** Agata Kołkowska, Joanna Michalska, Rafał Zieliński, Wojciech Simka

**Affiliations:** 1 Department of Inorganic Chemistry, Analytical Chemistry, and Electrochemistry, Faculty of Chemistry, Silesian University of Technology, 44-100 Gliwice, Poland; agatkol653@student.polsl.pl (A.K.); joanna.k.michalska@polsl.pl (J.M.); 2 Chemistry Students Research Society, Faculty of Chemistry, Silesian University of Technology, 44-100 Gliwice, Poland; 3 Stomatologia na Ksiezym Mlynie, 16D Tymienieckiego, 90-365 Lodz, Poland; bkost@op.pl

**Keywords:** electropolishing (EP), titanium alloys, dental implants, methanol, surface treatment, biomaterials

## Abstract

This paper reports the results of our study on electrochemical polishing of titanium and a Ti-based alloy using non-aqueous electrolyte. It was shown that electropolishing ensured the removal of surface defects, thereby providing surface smoothing and decreasing surface roughness. The research was conducted using samples made of titanium and Ti_6_Al_4_V alloy, as well as implant system elements: implant analog, multiunit, and healing screw. Electropolishing was carried out under a constant voltage (10–15 V) with a specified current density. The electrolyte used contained methanol and sulfuric acid. The modified surface was subjected to a thorough analysis regarding its surface morphology, chemical composition, and physicochemical properties. Scanning electron microscope images and profilometer tests of roughness confirmed significantly smoother surfaces after electropolishing. The surface profile analysis of processed samples also yielded satisfactory results, showing less imperfections than before modification. The EDX spectra showed that electropolishing does not have significant influence on the chemical composition of the samples.

## 1. Introduction

In the 1880s, with the development of titanium implants similar to those used today, the number of tooth replacement surgeries increased [1]. Tooth gaps not only affect functionality but also aesthetic aspects. The most common cause of tooth loss in adults is periodontal disease, followed by less common causes such as trauma and developmental defects. The use of titanium or its alloys for implants usually results in success [2].

Titanium alloys and pure titanium are particularly common in dental implantology due to their low density, good mechanical properties, and corrosion resistance. In the industry, pure titanium is designated as grades 1–4, differing in its purity, oxygen content, and physicochemical properties such as ductility, corrosion resistance, and strength [3]. Grade 4 is most commonly used for implant production [1]. Titanium alloy Ti_6_Al_4_V (TAV), often used in implantology, is referred to as grade 5. Despite safety concerns and increasing interest in beta alloys, TAV implants are still in use [4,5]. Aluminum and vanadium ions from this kind of implant can enter the human body. Aluminum affects bone mineralization, potentially causing structural deficiencies, while vanadium is cytotoxic and allergenic [2,6,7,8,9]. Surface treatment is applied to limit ion penetration into the human body [2].

Contact with saliva, a corrosive medium containing Cl- and F- ions, requires implants to have high chemical resistance. Additionally, they are exposed to chemicals in oral hygiene products. The aggressive environment underscores the importance of improving corrosion resistance [10]. Implant system components are particularly susceptible to continuous microbial colonization starting from the moment of implantation and lasting for the entire implant lifetime. Smoothing the surface reduces bacterial adhesion, thus limiting microbiological corrosion [11].

Both ion release limitation and corrosion resistance can be influenced by electropolishing (EP), an electrochemical process that smooths surfaces without damaging the metal structure. In EP, the metal undergoes treatment as the anode immersed with the cathode in an electrolyte and subjected to a current, dissolving into metal ions and dissolving in the electrolyte. By removing the surface layer, the metal is polished and passivated [4,12,13]. The surface layer formed during mechanical processing typically has a deformed structure and deviates from the core metal’s physicochemical properties, often negatively impacting corrosion resistance by increasing the surface electrochemical activity. Electropolishing eliminates this layer, resulting in significantly improved surface characteristics and properties [14,15]. This method does not cause mechanical interaction, damage, or residual stress [16]. Considering TAV electropolishing, an important issue is the dissolution of aluminum and its alloys being accelerated by the flow of an electric current in some solutions, especially acids. Sulfuric acid causes the dissolution of porous anodic oxide on aluminum, and its rate increases with time [17,18].

During the process, three chemical reactions occur simultaneously: anode dissolution, oxygen evolution and the formation of a passive oxide film. The first reaction describes how titanium atoms dissolve from anode parts, and the oxidation (passivation) reaction forms a thin and uniform TiO_2_ layer on the part surface, limiting the titanium anion diffusion into the electrolyte solution. Equations (1)–(3) present the equations of the reactions occurring during electropolishing of titanium [19,20,21].

Anode dissolution: Ti → Ti^4+^ + 4e (1)

Oxygen evolution: 4 OH^−^ → O_2_ + 2 H_2_O + 4e^−^ (2)

Formation of a passive oxide film: Ti + 2 OH^−^ → TiO_2_ + H_2_O + 2e^−^ (3)

The resulting oxide layer is unfavorable, and its dissolution is necessary for the continuation of the ongoing process. Initially, the voltage increases, and polishing takes place on a macro-scale. A sort of equilibrium is then established, allowing the top layer of metal to dissolve simultaneously and the oxide to form on its surface. The surface is then smoothed both on a macro-scale and polished on a micro-scale. This stage is the most important and determines the final morphology of the layer. The parameters used—voltage, temperature, and time—can significantly influence the process itself. The presence of water in the electrolyte used for EP significantly increases the probability of forming a passive layer on the metal surface. The efficiency of the electrolyte without water is therefore much greater, and the resulting surface is more shiny and polished [19,20].

In the literature, the most often described electrolytes are as follows:

- Fluoric acid-based electrolytes: fluoric acid, sulfuric acid, and acetic acid [22];
- Perchloric acid-based electrolytes: perchloric acid, methanol, and n-butanol [23]; perchloric acid and acetic acid [24]; and perchloric acid, methanol, and ethylene glycol [25];
- Perchloric acid-free electrolytes: methanol and sulfuric acid [26]; ethylene glycol and NaCl [27];
- Deep eutectic solvents (DESs) such as choline chloride and ethaline [28,29].

The electrolyte used in electropolishing also includes additives such as tri-ethanolamine (TEA), triethylamine (TRE), ethanolamine (MEA), diethanolamine (DEA), glycerol (GLR), and diethylene glycol monobutyl ether, known as butyldiglycol (BDG) [30].

The water content in the electrolyte has a huge impact on the entire electropolishing process. By limiting its amount using organic ingredients, you can significantly change the course of the process and specifically cause the limiting current density increase. According to research, 10% water content causes passivation of the anode at a wide range of potentials, impeding polishing. The latest findings reveal that electropolishing titanium in methanol–sulfuric acid electrolytes is controlled by mass transport. Variations in the current density suggest that transferring dissolved tetravalent titanium species from the anode surface limits the rate. High-frequency impedance data confirm this, indicating a compact salt film at the anode surface during current limiting [25,31].

The use of non-aqueous solutions is considered an alternative to acidic solutions, because the absence of water can significantly reduce the formation of a stable titanium oxide layer. Fluoride baths, used not only for electropolishing titanium but also other metals such as niobium, are limited due to their high acidity and environmental harm. Traditional baths pose risks when used in medical applications, as there is a risk of adverse substances entering the human body [20,32,33,34]. 

The use of sulfuric acid and methanol as an electrolyte has many advantages over other organic substances. Similarly to the use of deep eutectic solvents (DESs), an electrolyte consisting of sulfuric acid and methanol is easy to prepare. The proposed electrolyte is ready for use immediately after mixing, while ethaline, as an example of DES, must first be homogenized at an elevated temperature. High hygroscopicity causes the easy absorption of water by a DES, which has a huge impact on the electrochemical and physicochemical properties, so its content in the electrolyte should be constantly monitored. The low concentration of sulfuric acid and the biodegradable properties of methanol mean that there is no threat to the environment. The decisive factor in selecting the appropriate electrolyte is often economics. The price of choline chloride is eight times higher than that of H_2_SO_4_. Despite the need to use lower temperatures, due to the risk of overheating the methanol, using a H_2_SO_4_-based electrolyte is way cheaper than a DES; hence, more companies would be interested in this kind of solution [29,35,36].

Therefore, research has been undertaken on non-aqueous H_2_SO_4_-based electrolytes for electropolishing. The main purpose of the research is obtaining a smooth surface without causing a significant change in the chemical composition of the metal. 

## 2. Materials and Methods

Titanium (grade 4) and Ti_6_Al_4_V (grade 5) alloy samples in the shape of a cylinder with a diameter of 6 mm were used to select the appropriate parameters for the electropolishing process. The Ti_6_Al_4_V elements intended for dental implantology were then electropolished: analogues, healing screws, and multiunits. Before the process, the samples were degreased using ultrasound in isopropanol and dried in air.

The electropolishing process was carried out in a glass electrolyzer with diaphragm cooling. The cell was powered by a Kikusui PWR 800H high-voltage power supply (Kikusui, Yokohama, Japan). The temperature of the electropolishing bath was maintained at −20 °C using a cryostat. A magnetic stirrer was used for good heat and mass transfer. The samples constituting the anode were mounted using a dedicated anode holder. Acid-resistant steel in the form of a sheet adapted to the electrolyzer was used as the cathode. The ratio of the cathode to anode area was 250:1. As an electrolyte, an 8% H_2_SO_4_ solution in methanol was used. EP was carried out using three different limiting voltages: 10 V (EP1), 12.5 V (EP2), and 15 V (EP3). After 10 min, the samples were removed from the electrolyzer and immediately rinsed in methanol, then in demineralized water, and air-dried. Dry samples were submitted for testing. The sample labels are presented in Table 1.

### 2.1. Surface Morphology Analysis

The surfaces of all samples were thorough analyzed for their morphology and chemical composition with the use of a scanning electron microscope (SEM) with an energy dispersive X-ray spectrometer (EDX) before and after EP (Phenom ProX; ThermoFischer Scientific, Waltham, MA, USA). Analysis of the surface morphology is possible by scanning the sample surface with a beam of electrons, which are emitted by the cathode and then formed into a beam in the optical system. The signal emitted by the electrons is then processed, which allows for obtaining an SEM image. The formation of topographic contrast is related to the emission of secondary electrons. An accelerating voltage of 15 kV was used for the measurement. 

### 2.2. Wettability

The surface contact angle of the electropolished samples was also determined by us-ing the OCA15 goniometer (DataPhysics Instruments, Filderstadt, Germany). The test involves placing a drop of distilled water with a volume of 0.2 µL on the sample surface using a needle and then determining the wettability angle: the angle formed between the sample surface and the tangent to the drop. The presented result for the static contact angle is an arithmetic mean of 5 measurements for each sample. The dynamic contact angle determines the change in the wetting angle over time. The result has an accuracy of 0.01°.

### 2.3. Roughness

Additionally, a surface profile analysis was performed, from which the Ra and Rz (µm) values were read. Ra is the arithmetic mean of the profile ordinates, while Rz is the highest height or roughness selected from the 10 highest profiles measured. The influence of electropolishing on the roughness of samples was examined with the Prolilometer Mitutouo SJ301 (Mitutoyo S-301J, Kanagawa, Japan). This measurement involves moving a diamond blade vertically over the surface of the sample attached to the stand. The movement of the blade is converted into electrical signals and displayed on the screen in digital form. The result has an accuracy of 0.01 μm.

## 3. Results and Discussion 

The electropolished samples were first subjected to a visual surface evaluation. Special attention was paid to irregularities, pits, and surface uniformity. Subsequently, the samples underwent a detailed analysis to select appropriate parameters that would allow for obtaining layers with the best characteristics.

### 3.1. Surface Morphology

**Figure 1:**
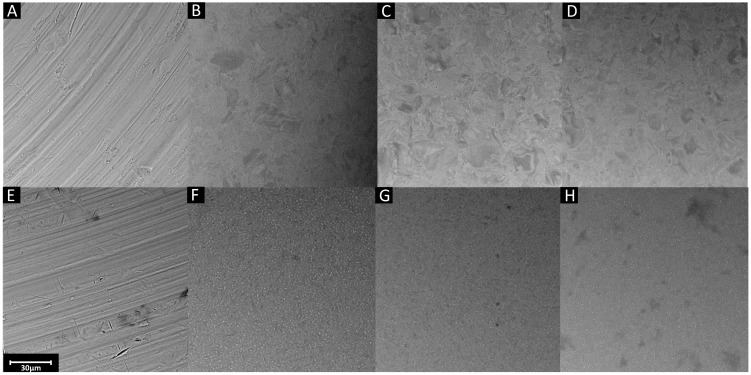
SEM images of (**A**) Ti-M, (**B**) Ti-M-EP1, (**C**) Ti-M-EP2, (**D**) Ti-M-EP3, (**E**) TAV-M, (**F**) TAV-M-EP1, (**G**) TAV-M-EP2, and (**H**) TAV-M-EP3.

The images from the scanning electron microscope before and after electropolishing are presented in Figure 1.

Figure 1A,E show SEM images of the reference titanium and Ti_6_Al_4_V alloy samples. The scratches resulting from grinding are deep and clearly visible. The surfaces of all samples after surface treatment are noticeably smoothed, with no visible scratches or irregularities. Electropolished titanium differs from Ti_6_Al_4_V alloy. In the SEM images of titanium, crystal boundaries are visible, which were exposed due to the dissolution of the surface layer. Throughout the surface of the Ti_6_Al_4_V alloy, uniformly distributed white points are visible, highlighting the alloying components. The crystal structure is best visible on the surface of the Ti-M-EP2 sample. A properly selected voltage allows us to obtain the best results. If the voltage is too low, the structure is not as polished as possible. Better results were obtained by changing the parameters. Using a voltage of 12.5 V, the best layer morphology is observed. Increasing the voltage did not improve the characteristics of the surface; on the contrary, the crystals on the titanium samples are less distinct. Considering the mechanism and reactions occurring during the process, too low and too high voltages could lead to a higher reaction rate of the formation of a passive film on the surface than the dissolution of the anode. The faster-forming layer blocks the possibility of further smoothing the surface, so the phase crystals are not as visible. Surface roughness affects the corrosion resistance of metals and their alloys. The smoother the surface, the greater the corrosion resistance. The absence of unevenness and the evenness of the surface especially prevent pitting corrosion. Taking this into consideration, the samples electropolished at 12.5 V may be the least exposed to corrosion [37,38,39].

**Figure 2:**
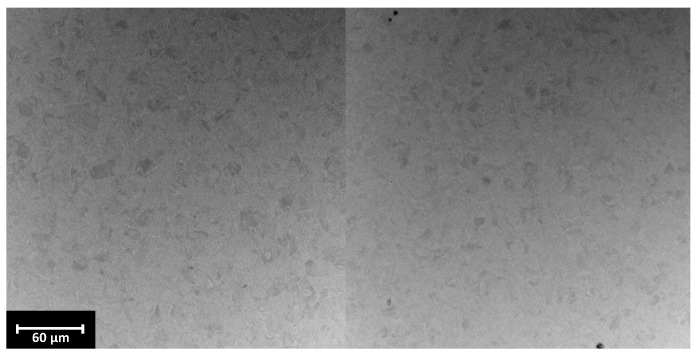
SEM images of two different Ti-M-EP2 samples.

The surfaces of samples electropolished using the same set of parameters have similar morphology, as shown in Figure 2 by comparing an example of SEM images of two different samples.

Both samples have the same characteristics, and the surface appears equally even. This proves the repeatability of the tests and the possibility of reproducing them, obtaining the same results.

**Figure 3:**
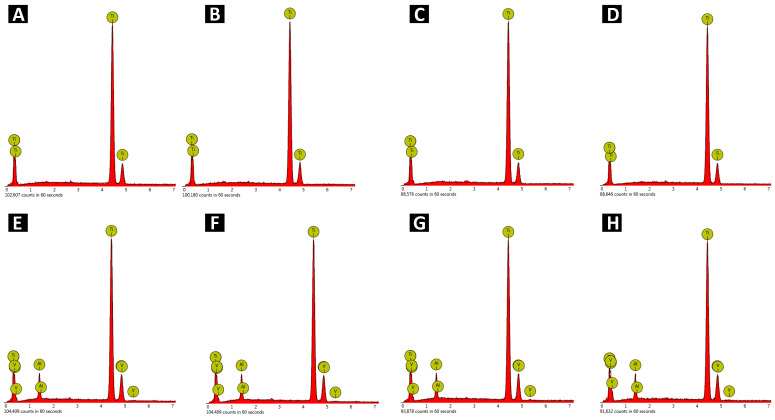
EDX spectra of (**A**) Ti-M, (**B**) Ti-M-EP1, (**C**) Ti-M-EP2, (**D**) Ti-M-EP3, (**E**) TAV-M, (**F**) TAV-M-EP1, (**G**) TAV-M-EP2, and (**H**) TAV-M-EP3.

To determine the chemical composition of the surface, EDX analysis was performed (Figure 3).

EDX analysis confirmed that the samples are chemically pure and that there are no contaminating elements on their surfaces. Electropolishing has an unnoticeable effect on changing the chemical composition of the samples. Unlike classic solutions, no particles from the electrolyte are detected on the surface of the tested samples, which allows for the conclusion that they are safe for use in contact with the human body [20]. The changes concerned only the topography of the samples, not the chemical composition. 

**Figure 4:**
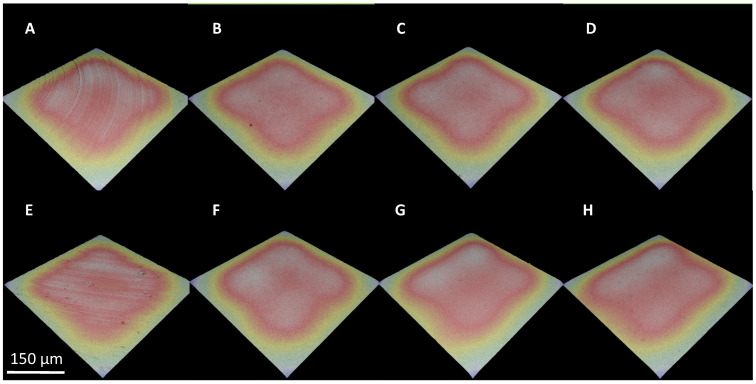
3D mapping of (**A**) Ti-M, (**B**) Ti-M-EP1, (**C**) Ti-M-EP2, (**D**) Ti-M-EP3, (**E**) TAV-M, (**F**) TAV-M-EP1, (**G**) TAV-M-EP2, and (**H**) TAV-M-EP3.

In order to precisely analyze the irregularities present on the sample surface, 3D mapping was performed (Figure 4).

The 3D mapping confirmed the changes noticed during SEM analysis. All samples have been smoothed, and scratches after grinding are not noticeable. There are no irregularities on the surface, and its roughness has visibly decreased. There are no significant differences between all electropolished samples. All of them appear similar to each other. To confirm or reject the conclusions drawn, an additional roughness analysis was performed.

### 3.2. Roughness

The roughness was measured using two different methods: with a profilometer at the macro-scale and using 3D mapping at the micro-scale. Table 2 presents a summary of the Ra and Rz values, determining the change in roughness before and after electropolishing at micro- and macro-scales. The Ra value determines the arithmetic average deviation from the mean line, while Rz is the highest roughness height according to the 10 highest profiles measured. Additionally, Sa is shown, which describes the difference in the height of each point compared to the arithmetical mean of the surface.

Smoothing the surface during electropolishing decreased the roughness of the surface. The values of both Ra and Rz for modified samples are much lower than for the reference samples, as expected. The Sa values for titanium decreased due to electropolishing. In the case of TAV, EP1 and EP3 increased the parameter value, while EP2 decreased it. The values of both parameters for titanium are lower than for TAV. At the macro-scale, the titanium sample electropolished at 12.5 V (EP2) has the lowest roughness, while for the TAV alloy, electropolishing at 15 V (EP3) turned out to be the most effective. The values obtained for the previously mentioned samples are almost identical. At the micro-scale, all of the Ra values are similar after electropolishing. There is a huge difference between the reference samples and modified samples. The result obtained for the reference sample is over two times bigger. The Rz result is the lowest for the macro-scale for the Ti-M-EP2 and TAV-M-EP3 and for the micro-scale Ti-M-EP3 and TAV-M-EP1. The literature data confirm the decreasing tendency of the Ra and Rz parameters during electropolishing [4,40,41].

### 3.3. Contact Angle

The static contact angle is shown in Table 3. From the obtained results, it is difficult to determine the effect of electropolishing on the contact angle.

Considering titanium, one of the samples (Ti-M-EP1) is characterized by a much smaller contact angle. For the Ti-M-EP2 sample, the contact angle is close to the value for the reference sample, while for Ti-M-EP3, the wettability increased. For the Ti_6_Al_4_V alloy, the samples electropolished at 10 V and 12.5 V increased their wettability. For the sample subjected to the EP process at 15 V, a decrease in the wetting angle was observed. The lack of correlation in the values obtained during the test does not allow for a proper description of the impact of electropolishing on the contact angle. According to the literature, as the roughness decreases, the contact angle increases [40]; hence, the best electropolishing result should be characterized by the highest hydrophilicity. Taking into account the above assumption, the best result is EP3 for titanium and EP1 for TAV. Due to the relatively high inaccuracy of the method and the lack of meaningful relationships between the results, other research results were used.

**Figure 5:**
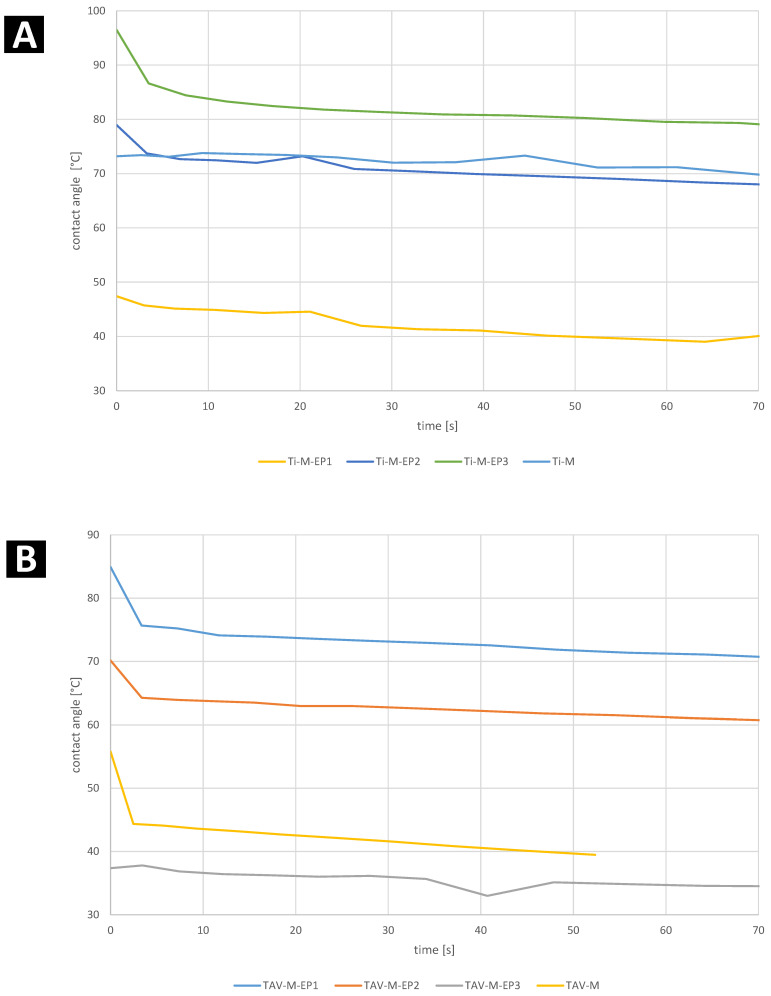
Dynamic contact angle for (**A**) titanium and (**B**) Ti_6_Al_4_V samples.

Figure 5 show graphs of the dependence of the wetting angle on time. 

As with the static contact angle, the data are inconclusive. The wettability of all samples stabilizes very quickly. After 10 s, the function becomes almost constant, and the drop no longer spreads on the sample surface. The first part of the function for TAV declines more rapidly than for titanium.

### 3.4. Implant System Element Electropolishing

**Figure 6:**
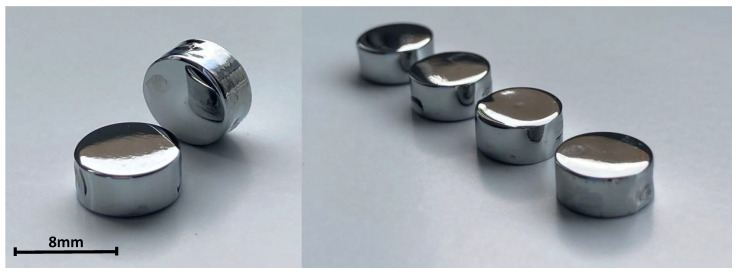
Macro-image of the titanium samples after EP.

**Figure 7:**
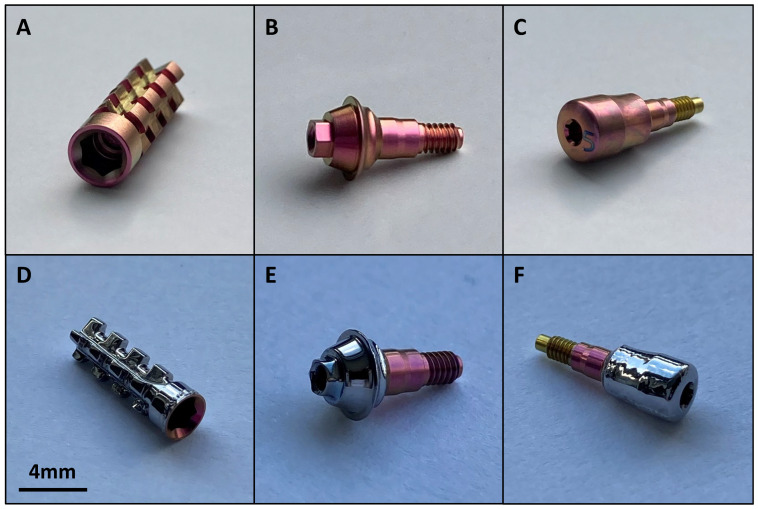
Macro-image of the Ti_6_Al_4_V samples before EP: (**A**) analogue, (**B**) multiunit, and (**C**) healing screw; after EP: (**D**) analogue, (**E**) multiunit, and (**F**) healing screw.

Based on the analyses performed, electropolishing method No. 2 (12.5 V, 10 min) was found to be the most effective. The elements of the implant system were processed using selected parameters. Figure 6 and Figure 7 show macro-photographs of electropolished samples.

Electropolishing allowed us to obtain a smooth surface, with imperfections invisible to the naked eye. The samples shine, resembling a mirror.

The electropolishing process using the developed parameters was successfully replicated on real elements of the implant system. EP-treated fragments visibly differ in both color and structure. The pink layer resulting from anodizing was dissolved, and the surface became smooth and shiny. The screw threads were secured during the process. Due to a slight loss of volume, the electropolished thread might not fit to the other elements or be loose. This creates a risk of the connections loosening and, as a result, falling out.

**Figure 8:**
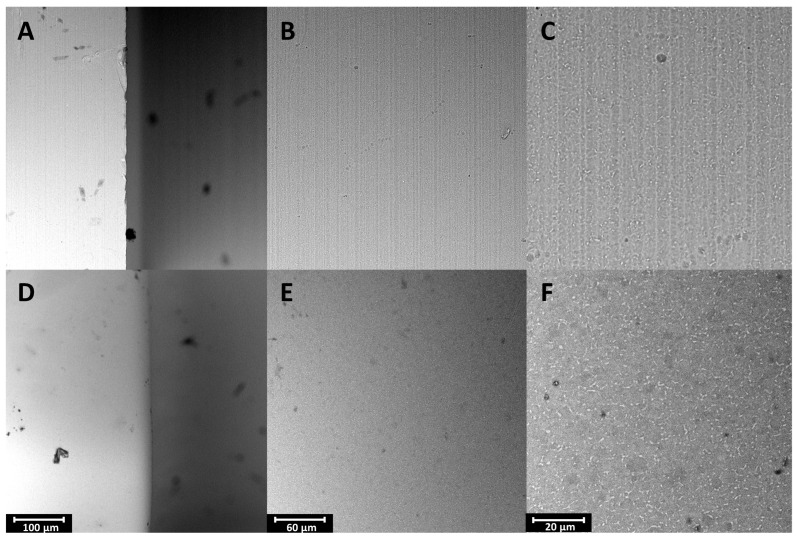
SEM images of the analogue before (**A**–**C**) and after EP (**D**–**F**).

In order to thoroughly analyze the surface and check whether its morphology is analogous to flat samples, SEM images were taken (Figure 8).

The transfer of parameters from the test samples to actual components of the implant system was successful. The surface after EP is smooth and has no visible irregularities on a micro-scale. It is similar to the surfaces obtained for TAV samples. Figure 8A,D compare the edge of the analogue before and after EP, which is visibly rounded as a result of polishing. After the process, the previously present nicks and irregularities disappeared. The lines remaining after the production of the analogue (Figure 8B,C) have been smoothed, and the individual components of the alloy are even more visible after EP (Figure 8E,F). 

## 4. Conclusions

As a result of electropolishing, a smooth surface was obtained on samples made of titanium and the Ti_6_Al_4_V alloy. An analysis with an SEM microscope allowed us to understand the surface characteristics of the samples. All the parameters used led to a significant smoothing of the surface. The best result was obtained using a voltage of 12.5 V. No changes in the chemical compositions of the samples and no contamination were confirmed using energy dispersive X-ray spectrometer analysis. The 3D mapping showed the removal of grinding scratches on each of the processed samples. EP allowed for the reduction of the Ra and Rz values for all parameters used. The lowest value of Ra = 0.09 nm was obtained for titanium electropolished at 12.5 V and for TAV electropolished at 15 V. The results of the contact angle analysis were unclear. A significant decrease was observed for titanium, while for TAV, there was an increase in the contact angle value. To consider them reliable, the analysis should be repeated. Based on the conducted research, EP2 electropolishing was selected as the one that best meets the expectations, and these parameters were used for processing the elements of the implant system. Satisfactory results were obtained, and a smooth surface on each sample was clearly observed. 

## Figures and Tables

**Table 1:** Sample labels.

Sample Label	Surface Modification
Ti-M	Titanium reference sample
Ti-M-EP1	Titanium electropolished, 10 V, 10 min
Ti-M-EP2	Titanium electropolished, 12.5 V, 10 min
Ti-M-EP3	Titanium electropolished, 15 V, 10 min
TAV-M	Ti_6_Al_4_V reference sample
TAV-M-EP1	Ti_6_Al_4_V electropolished, 10 V, 10 min
TAV-M-EP2	Ti_6_Al_4_V electropolished, 12.5 V, 10 min
TAV-M-EP3	Ti_6_Al_4_V electropolished, 15 V, 10 min

**Table 2:** Ra, Rz, and Sa results before and after electropolishing Ti and Ti_6_Al_4_V.

Sample Label	Ra, μm		Rz, μm		Sa, μm
	Micro-Scale	Macro-Scale	Micro-Scale	Macro-Scale	
Ti-M	0.685 ± 0.070	0.96 ± 0.23	4.26 ± 0.56	5.83 ± 1.38	0.763
Ti-M-EP1	0.276 ± 0.020	0.52 ± 0.27	1.52 ± 0.10	2.39 ± 1.73	0.385
Ti-M-EP2	0.287 ± 0.043	0.09 ± 0.03	1.47 ± 0.07	0.49 ± 0.13	0.443
Ti-M-EP3	0.261 ± 0.025	0.13 ± 0.06	1.35 ± 0.09	1.10 ± 0.96	0.403
TAV-M	0.566 ± 0.079	0.61 ± 0.04	3.03 ± 0.41	2.80 ± 1.44	0.497
TAV-M-EP1	0.227 ± 0.046	0.68 ± 0.29	1.31 ± 0.22	2.59 ± 0.91	0.592
TAV-M-EP2	0.275 ± 0.021	0.36 ± 0.11	1.50 ± 0.08	2.51 ± 1.25	0.406
TAV-M-EP3	0.262 ± 0.050	0.09 ± 0.04	1.33 ± 0.20	0.47 ± 0.12	0.566

**Table 3:** Static contact angle of titanium and Ti_6_Al_4_V samples.

Sample Label	Contact Angle [°C]	Standard Deviation [°C]
Ti-M	73.85	2.26
Ti-M-EP1	32.26	6.79
Ti-M-EP2	72.02	2.74
Ti-M-EP3	81.28	5.00
TAV-M	51.86	3.43
TAV-M-EP1	80.49	4.61
TAV-M-EP2	78.08	2.76
TAV-M-EP3	39.06	3.15

## Data Availability

The data presented in this study are available on request from the corresponding author. The data are not publicly available due to company confidentiality.
